# Chinese Minority Perceives the Doctor-Patient Relationship Differently: A Cultural and Economic Interpretation

**DOI:** 10.3389/fpubh.2019.00330

**Published:** 2019-11-18

**Authors:** Qian Yang, Hao Zhang, Mengfei Yu, Xiaoqian Hu, Yuxuan Gu, Xueshan Sun, Xuemei Zhen, Shuyan Gu, Minzhuo Huang, Jingming Wei, Yuhang Zeng, Hengjin Dong

**Affiliations:** ^1^Center for Health Policy Studies, School of Public Health, Zhejiang University School of Medicine, Hangzhou, China; ^2^Department of Social Medicine, School of Public Health, National Clinical Research Center for Child Health of The Children's Hospital, Zhejiang University School of Medicine, Hangzhou, China; ^3^School of Medicine, Hangzhou Normal University, Hangzhou, China; ^4^Department of Social Medicine, School of Public Health, The Fourth Affiliated Hospital Zhejiang University School of Medicine, Yiwu, China

**Keywords:** doctor-patient relationship, socioeconomic status, ethnic minorities, medical sociology, social change, hospital hierarchy

## Abstract

In China, doctor-patient relationships (D-P relationships) are cited frequently and attracted international attention. This study assesses whether the D-P relationship experienced by the Chinese is associated with ethnicity, hospital hierarchies, and socioeconomic factors. In a national cross-sectional survey, multi-stage random sampling was adopted to assess regional and socio-economic differences between year 2016 and year 2017. Each area surveyed consisted of about 250 randomly chosen households, and valid results were obtained from 4,173 adults aged 16–99. When provided a choice of eight types of D-P relationship, for doctors in primary care institutions, 63.8% of ethnic minorities indicated having a friend-type relationship with their physicians, with 23.3% having a trading/reciprocal relationship. Han Chinese, however, predicts the opposite relationship between doctors from different hierarchy and the types of D-P relationship. For physicians working in hospitals, this difference in relationship was more pronounced, where 52.9% of ethnic minorities indicated having a friend-type relationship with their physicians, and 30.1% indicated the presence of a trading/reciprocal relationship. For Han Chinese, however, 53.3% indicated having a reciprocal relationship with their doctor. Overall, the prevalence of friendly D-P relationships was correlated with ethnic minorities, lower levels of education, and lower incomes. Ethnic minorities are most likely to perceive their physicians as friends, while Han Chinese are more likely to perceive a trading relationship with their physicians. The primary contribution of this research is the finding that D-P relationships differ for Han Chinese and other ethnic minorities.

## Introduction

Chinese doctor-patient relationships (D-P relationships) have been scrutinized intensely in recent years. Violence against doctors (VAD) has been frequently reported both domestically and abroad. D-P relationships in China have been characterized by an increasing number of medical disputes and even outright violence (1, 2). A report published in 2015 by the Chinese Medical Doctor Association reported almost 60% of medical staff in China have experienced verbal abuse and around one in seven have been physically assaulted (3).

Traditionally, the Chinese have had more positive D-P relationships. In ancient China, people greatly respected physicians. One story tells of a seriously ill duke two thousand years ago who asked for the services of a physician named Hwan. Before Hwan came, the duke dreamt that his disease turned into two boys, who said, “That is a skilled physician; it is feared he will hurt us; how shall we get out of his way?” Then, one of them said, “If we take our place above the heart and below the throat, what can he do to us?” When the physician arrived, he said, “Nothing can be done for this disease. Its seat is above the heart and below the throat. If I assail it with medicine, it will be of no use; if I attempt to puncture it, it cannot be reached. Nothing can be done for it.” If this happened today, the physician might be attacked by a patient who values utility first and foremost. However, the duke said, “He is a skilled physician, to give him large gifts, and to send him back to his hometown” (4).

The correlation between D-P relationships and race or ethnicity is also a worldwide concern. A study comparing perceived D-P relationships among different ethnicities in Israel showed that Arabs more frequently chose the term “friend” to describe their current D-P relationship than Jews, and generally desired this friend relationship (5). While no study has been conducted on perceptions of D-P relationships in China, Chinese ethnic minorities have been found to have different cognition and behaviors than that of Han Chinese, including different cultural histories, ecologies (6), and health practices. For instance, Chinese ethnic minorities experience different medical resource distributions, health equity (7), and mental health prejudices (8). China officially recognizes 55 ethnic minority groups in addition to the Han majority (9). According to the sixth Census of Population, the Han population consisted of 1225.9 million people, accounting for 91.51%; while the ethnic minority population consisted of 113.8 million people, and only 8.49% (10). Folk psychological studies have found that the ethnic minorities have more pronounced emotions than Han Chinese (11). In addition, ethnic minority students are more satisfied with neighborhood relationships than the Han Chinese (12).

While China was undergoing globalization, modernization and urbanization, the differences between the ethnic minorities and the Han Chinese were more pronounced. Before reform, China was characterized by collectivism and Confucian dynamics (13–15). Its predominant features were persistence (perseverance), personal steadiness (reliability), respect for tradition, and reciprocation (16). It has been argued that the transition to a market economy has led to the moral deterioration of Chinese citizens. The development of business ethics and professional morality has not kept pace with market reforms (17–19). Lack of professionalism within the Chinese health care system has also been considered one of the main problems resulting from marketing and medical reforms (20).

Conversely, it has been found that income has less impact on the well-being of ethnic minorities than that of the Han Chinese (11). It seems that Confucianism, which values respect for brotherhood, social harmony, and protection of the interests of one's in-group (21, 22), has been retained more strongly by ethnic minorities. One of the significant expressions of this trend in the Han Chinese is the loss of trust among interpersonal relationships that is vital to health care reform.

A discussion of Chinese relationships is critical to Chinese indigenous psychology. The representative theories of Chinese indigenous psychology are based mostly on a pattern of difference sequence (23, 24), which describes the essence of social structure and relationship in an earthbound community in China. Like the concentric waves that become bigger and bigger after a stone is thrown into the water, every person is in the center of his own circle, and his or her social relationships emanate outwards. The Chinese trust people closer to the center (self), like family and friends, and put less trust in people further away from them, such as customers or strangers. This may influence patients' attitudes toward their doctors based on which layer in which patients put their doctors. Because Chinese indigenous psychology has yet to provide an answer as to which layer this may be, we refer to western medical knowledge concerning D-P relationships.

Generally speaking, the D-P relationships have experienced three revolutions in western culture. Initially, in 1847, the American Medical Association Code of Medical Ethics called for “total patient obedience.” Gradually, a respect for patient autonomy began to form (25). Until the D-P relationships were transformed from a traditional paternalistic style (26) to a buyer and seller relationship (27). For example, the Patient Protection and Affordable Care Act (also known as Obamacare) in the US referred to the “consumer” instead of the “patient” (28). However, this has been repudiated in a recent study (5), marking a third transformation. The World Health Organization (WHO) and its international declarations articulated people-centered healthcare approaches (29). These approaches advocate for the knowledge of a patient's value, and for “treating the whole patient,” with attention to therapeutic relationships (30–33).

According to studies of D-P relationships in various cultures, D-P relationships can be described as client, customer, insured, partner, or friend relationships (25, 34–36). Given that *friend* is a term that infers trust, interest, and a more human connection, those who perceive that they have relationships similar to friendships with their doctors are more likely to have confidence in their treatment. In part, confidence in one's treatment is a measure of confidence and trust in one's physician. Studies of patient satisfaction show the importance of “being known,” “having a human connection,” and “being understood” as intrinsically valuable (37, 38).

The functions of hospitals and primary health institutions are different. Doctors in community centers have more primary care responsibilities, which include first-contact access for each new need, long-term person-focused care (as opposed to disease-focused), comprehensive care for most health needs, and coordinated care in referrals (39). The nature of primary care suggests that doctors who work in primary care institutions may differ from doctors who work in hospitals, in that they tend to have a pro-social orientation toward family and community (40). In contrast, Chinese public hospitals have faced policy and regulation problems because social responsibilities are unfunded and, thus, hospital directors are preoccupied with generating revenue from services and medicines to cover basic operational costs (41).

Based on these differences in the focus of hospitals and primary care institutions, D-P relationships also differ between hospitals and primary care institutions. Several studies have compared D-P relationship perspectives of Chinese medical staff in urban tertiary hospitals and primary care institutions. The results consistently showed that medical staff in higher-level hospitals scored lower on harmony of the D-P relationships, while doctors and nurses in primary care institutions gave higher scores (42–44).

This study tested this conclusion and the impact of ethnicity on the perception of D-P relationships by comparing two representative provinces in the eastern and western regions of China. These two regions were chosen because ethnic minorities are more concentrated in western China, with 46 ethnic minorities represented, while Han Chinese predominantly live in eastern regions. All five ethnic autonomous regions in the country are in the west, with an area of 5.95 million square kilometers, accounting for 85.95% of the western total area. According to the Confucian teaching, *courtesy calls for help from the wild* (礼失而求诸野) (45), the lesser impact of social transitions on minority groups may be the result of the preservation of traditional Chinese culture and values, including trust in and friendly perceptions of physicians. Thus, we surveyed residents both in Zhejiang and Qinghai to compare patient perspectives on D-P relationships in big hospitals and primary care health institutions.

## Method

### Study Design and Participants

This cross-sectional study is part of a national project studying referral systems. The survey assessed satisfaction with health care services and was composed of six parts: general family conditions, the individual conditions of family members, illnesses, and injuries occurring within the last 2 weeks, hospitalizations in the previous year, satisfaction with and accessibility of primary health care institutions, and willingness to receive health care services, with the latter being the focus of variable assessment in the survey section. Two provinces were selected as the main study sites: Zhejiang Province and Qinghai Province. Since 2016, Zhejiang and Qinghai provinces have been identified as the new medical health system reform pilot provinces by the National Health Commission of the PRC (the former National Health and Family Planning Commission), they play a demonstration effect in the exploration of medical and health system optimization and hierarchical diagnostic model. More importantly, Zhejiang Province is a wealthy eastern province neighboring Shanghai City, while Qinghai Province is an inner province of northwestern China. Thus, it has a much slower speed of globalization, modernization, and urbanization than Zhejiang province. Investigations were conducted during 2016 (GDP [in billions of ¥] of Zhejiang: 4,725,136; Qinghai: 257.25) and 2017 (GDP of Zhejiang: 5,176,826; Qinghai: 262.48). The ethnic minority population in Qinghai province is 46.98%, but in Zhejiang, there are only 2.23% (according to the latest census data of China). By choosing Qinghai and Zhejiang as the study sites, we could compare the ethnic minorities and Han Chinese conveniently.

The survey was conducted among the resident population in each region, including the residents moving to the area in the past 6 months and those living there for more than 6 months. The exclusion criteria were those under 16 years old and those could not response by themselves, such as those work outside of the county or district. Their data were reported by their parents. Thus, our data still covered the situation of all ages. According to principles of purposiveness, economic efficiency, and feasibility, the multi-stage probability sampling was used. In the first stage, Qinghai and Zhejiang provinces were selected. In the second stage, we have adopted the multistage clustered probability sampling frame. There are 33 counties in Zhejiang and 34 counties in Qinghai. After running a Q-cluster analysis method in terms of 10 socio-economic development indices and 5 key indicators of health system development, we have found that Zhejiang has 2 layers (developed and undeveloped), and Qinghai has 3 layers (developed, developing and undeveloped). Thus, in Zhejiang Province, the representative regions were Jiashan County (a developed region) and Jinyun County (an undeveloped region). In Qinghai Province, we sampled four counties or districts because the population density in Qinghai Province is much lower (8 persons/km^2^) than in Zhejiang Province (533 persons/km^2^) (46). We sampled Chengxi district of Xining city (developed), Ping'an district of Xining city Huzhu county (developing), and Jianzha county (undeveloped) in Qinghai. Names of the counties and number of households selected in both Provinces were in Table 1. In the third stage, Probability Proportionate to Size Sampling (PPS) was used in Qinghai province. The calculation indices that proportion of permanent resident, urban population and rural population in developed, developing and undeveloped layers were presented in Table 2. The calculation indices of Zhejiang province are the proportion of agricultural and non-agricultural population instead of the proportion of urban and rural population. Because as an eastern province, the rate of urbanization of Zhejiang is very fast. In recent years, many agricultural populations have been divided into urban population, and the living habits and health service utilization habits of farmers living in cities are obviously different from those of real urban population. Although, Zhejiang urban population accounted for 67%, while the rural population accounted for 34%, but in 2016, Zhejiang province town reduced to 67%, which means, the non-agricultural population was only 32%, while the agricultural population reached to 68%. Therefore, the sampling according to the proportion of agricultural and non-agricultural population drawn with a more realistic representative than proportion of urban and rural population.

**Table 1:** Sampling distribution of residents' health service utilization.

**Province**	**County name**	**Representative characteristic**	**Street/town name**	**Sample size (household)**
Zhejiang	Jiashan county	Developed	Luoxing street	250
Zhejiang	Jiashan county	Developed	Yaozhuang town	250
Zhejiang	Jinyun county	Undeveloped	Wuyun street	250
Zhejiang	Jinyun county	Undeveloped	Xinjian street	250
Qinghai	Chengxi district	Developed	Pengjia stockaded village	276
Qinghai	Ping'an district	Developing	Ping'an town	100
Qinghai	Huzhu county	Developing	Donggou town	124
Qinghai	Jianzha county	Undeveloped	Cuozhou town	100

**Table 2:** The percentage of population characteristic of sampling sites.

**Population characteristic**	**Developed (%)**	**Developing (%)**	**Undeveloped (%)**	**Total (%)**
**The percentage of Qinghai resident's population**				
Permanent	47.93	37.36	14.71	100.00
Urban	33.06	12.31	4.41	49.78
Rural	14.87	25.05	10.31	50.23
**The percentage of Zhejiang resident's population**				
Permanent	47.93	–	14.71	100.00
Non-agricultural	26.87	–	4.93	31.80
Agricultural	45.36	–	22.83	68.20

### Data Collection and Quality Control

The data were collected by well-trained and qualified investigators through household survey and investigation. Investigators were recruited in the local study sites. They are young people with educational level equal or above high school. The investigators inquired all household members in local language according to the questionnaire survey items. They conducted the investigation when they were off work or in the weekends.

The content of the questionnaire and survey process were optimized and modified after consulting health policy research experts, health administrators, and medical staff. Over the course of the investigation, the objectives were clarified, and the survey specifications altered accordingly. When participants raised questions, the investigators gave immediate feedback. The informed consent was obtained from the participants before the survey. Graduate students from Zhejiang University were responsible for the data quality. They investigated each site for quality control, verification, and re-examination (sampling) after the survey. They also checked and verified the errors and omissions in the questionnaires each day during the survey.

### Dependent Variables

The indicators in this study are perceived D-P relationships in primary care institutions and perceived D-P relationships in big hospitals. The survey questions for each of these indicators were as follows.

→*Perceived D-P Relationships in Primary Care Institutions*: D-P relationships in primary care institutions are most similar to which of the following relationships? (1) parents and children, (2) teachers and students, (3) friends, (4) work partners, (5) comrades, (6) superiors and subordinates, (7) trading services/reciprocal, or (8) other.
→*Perceived D-P Relationships in Big Hospitals*: D-P relationships in big hospitals are most similar to which of the following relationships? (1) parents and children, (2) teachers and students, (3) friends, (4) work partners, (5) comrades, (6) superiors and subordinates, (7) trading services/reciprocal, or (8) others.

### Key Independent Variables

While we looked at individual and household social and economic characteristics (**Table 4**), the main independent variable we wanted to explore was ethnicity and the type of health care institution individuals sought first, which were assessed with the following questions.

*Ethnic Nationality*. “Please choose your ethnicity as below:” The top eight ethnicities found in the study areas were listed as the options: Han, Zhuang, Hui, Wei, Meng, Zang, Miao, and She. For analysis, ethnicity was dichotomized into Han Chinese and ethnic minority for simplicity. we coded the response of Han as “majority” and the rest as “ethnic minorities.”
*First Contact Health Institution*. We asked which kind of health institution respondents usually contact first, including six types of health care institution: (1) township health center, (2) community health center, (3) county/city/district of the provincial-level health institutions, (4) municipality/area/municipal district health institutions, (5) province/autonomous region/municipality directly under the central government health institutions and above, (6) others. For analysis, we coded responses of 3–5 as “big hospital” (0), 1–2 as “primary care institutions” (1), and 6 as “others” (2).

### Statistical Analysis

For statistical analysis, age was categorized into five groups: 18–29, 30–39, 40–49, 50–64, and 65+. Education level was separated into eight levels. Relative income was dichotomized to average or below average and above average. Because the per capita income of Qinghai Province (*Mean* = 11,779 ¥, *SD* = 24,454 ¥) is much lower than that of Zhejiang Province (*Mean* = 26,722 ¥, *SD* = 21,625 ¥), we calculated the mean of the two provinces separately. Based on an initial review of the survey results that indicated relatively low proportions for six of the eight types of relationships, perceived D-P relationships were categorized into friends, trading services, and other for doctors in primary care institutions and large hospitals. In accordance with social transition theory, the possible influence of ethnicity, the openness of different regions, and economic factors, which may have similarities to social transitions, have been controlled for their impact on the perception of D-P relationships. We also controlled the major demographics that may have confounding effects. Thus, the independent variables included ethnicity, province, income, education, sex, and age (**Tables 5**, **6**). A potential interaction was also considered: between province and ethnicity.

A chi-square test was used to analyze the association between demographic characteristics and perceived D-P relationships. A *p* < 0.05 was considered statistically significant. Population characteristics are described as percentage of the non-missing value. We also used a chi-square test to compare the difference of D-P relationships between Han Chinese and ethnic minorities according to different types of health care institutions (Table 3).

**Table 3:** Han and minority perceived D-P relationship in primary care institutions and large hospitals.

		**Han (*n*, %)**	**Minority (*n*, %)**	**Total (*n*, %)**	***p*-value**
Primary care institutions	Friend-type	1,093 (29.4)	293 (63.8)	1,386 (33.2)	
	Trading/reciprocal-type	1,470 (39.6)	107 (23.3)	1,577 (37.8)	
	Other-type	1,151 (40.0)	59 (12.9)	1,210 (29.0)	
	Total	3,714 (100.0)	459 (100.0)	4,173 (100.0)	<0.001
Large hospitals	Friend-type	477 (12.8)	243 (52.9)	720 (17.3)	
	Trading/reciprocal-type	1,987 (53.5)	138 (30.1)	2,125 (50.9)	
	Other-type	1,250 (33.7)	78 (17.0)	1,328 (31.8)	
	Total	3,714 (100.0)	459 (100.0)	4,173 (100.0)	<0.001

*p-value compared with Han and Minority by χ^2^-test*.

The dependent variable is the D-P relationships, it is a multi-way categorical dependent variable. Thus, multinomial logistic regression was performed (47). Multinomial logistic regressions with only one independent variable were used to calculate odds ratios (ORs). Adjusted odds ratios (AORs) were computed using multinomial logistic regressions with all independent variables entered simultaneously. All analyses were performed using the SPSS statistical software Version 18.0.

## Results

### Sociodemographic Characteristics

Sociodemographic analysis showed that there are significant differences in age, education, income, and province between ethnic minorities and Han Chinese. Gender distributions show no significant difference (Table 4).

**Table 4:** Sociodemographic characteristics of the sample.

	**Total (*n*, %)**	**Han (*****n*****, %)**		**Minority (*****n*****, %)**		***p*-value**
		**Zhejiang**	**Qinghai**	**Zhejiang**	**Qinghai**	
Age group (years)						<0.001
16–	217 (5.3)	98 (3.9)	82 (6.7)	1 (3.7)	36 (8.3)	
20–	585 (14.2)	292 (11.7)	223 (18.2)	6 (22.2)	64 (14.8)	
30–	669 (16.0)	387 (15.5)	181 (14.8)	11 (40.7)	90 (20.8)	
40–	926 (22.2)	518 (20.8)	303 (24.8)	4 (14.8)	101 (23.4)	
50–	1,087 (26.0)	708 (28.4)	288 (23.5)	3 (11.1)	88 (20.4)	
65–	689 (16.5)	488 (19.6)	146 (11.9)	2 (7.4)	53 (12.3)	
Subtotal	4,173	2,491	1,223	27	432	
Sex						0.82
Male	2,063 (49.5)	1,238 (49.7)	598 (48.9)	14 (51.9)	213 (49.3)	
Female	2,105 (50.5)	1,253 (50.3)	625 (51.1)	13 (48.1)	214 (50.1)	
Subtotal	4,168	2,491	1,223	27	427	
Education						<0.001
Never attend any school	467 (11.2)	152 (6.1)	167 (13.7)	1 (3.7)	147 (34.0)	
Primary school	942 (22.6)	645 (26.0)	216 (17.7)	5 (18.5)	76 (17.6)	
Junior middle school	1,291 (31.0)	787 (31.7)	404 (33.0)	12 (44.4)	88 (20.4)	
High school	474 (11.4)	270 (10.9)	156 (12.8)	3 (11.1)	45 (10.4)	
Technical school	65 (1.6)	35 (1.4)	24 (2.0)	2 (7.4)	4 (0.9)	
Secondary specialized school	173 (4.2)	122 (4.9)	41 (3.4)	0	10 (2.3)	
Junior college	394 (9.5)	233 (9.4)	127 (10.4)	1 (3.7)	33 (7.6)	
More than or equal to undergraduate degree	360 (8.6)	240 (9.7)	88 (7.2)	3 (11.1)	29 (6.7)	
Subtotal	4,166	2,484	1,223	27	432	
Per capita (¥/person/year)						<0.001
≤ 25%	999 (24.1)	661 (26.6)	176 (14.6)	13 (48.1)	200 (46.8)	
25% < ¥ ≤ 50%	1,174 (28.3)	584 (23.5)	332 (27.5)	6 (22.2)	121 (28.3)	
50% < ¥ ≤ 75%	1,123 (27.1)	502 (20.2)	359 (29.7)	5 (18.5)	52 (12.2)	
>75%	849 (20.5)	735 (29.6)	342 (28.3)	3 (11.1)	54 (12.6)	
Subtotal	4,145	2,482	1,209	27	427	
First contact health institution						
Big hospital	1,114 (44.7)	128 (10.5)	7 (25.9)	158 (36.6)		
Primary care health institution	1,363 (54.7)	1,094 (89.5)	20 (74.1)	274 (63.4)		
Other institution	14 (0.6)	1 (0.1)	0	0		
Subtotal	2,491	1,223	27	432		<0.001

*p-value compared with Han and Minority by χ^2^-test*.

Generally speaking, the Han Chinese have a higher socioeconomic status than ethnic minorities, with the first quartile of Zhejiang Province being equal to the fourth third of Qinghai Province, about 12,500 ¥. The first quartile of Qinghai Province is only 3,333 ¥, and the 2nd quartile is 6,667 ¥. The 2nd quartile of Zhejiang Province is 20,000 ¥ and the third quartile is 33,333¥.

### D-P Relationships

Respondents considered D-P relationships with primary care doctors to be similar to friendships (33.2%), trading services (37.8%), or other types (29.0%). However, for large hospitals, there was a strong association with business-type (trading) D-P relationships (50.9%), with few associating doctors in hospitals with friends (17.3%). This general pattern was observed in both Qinghai Province and Zhejiang Province.

The main finding is the effect of ethnicity on D-P relationships. The Han Chinese perceive D-P relationships differently from ethnic minorities (Table 3). For primary care doctors, 63.8% of the ethnic minorities indicated having a friend relationship with their physician, but only 23.3% indicated having a trading relationship. For the Han Chinese, 29.4% indicated a friend relationship, and 39.6% had trading relationships. However, for physicians working in big hospitals, 52.9% of the ethnic minorities indicated a friend relationship with their physicians, and 30.1% indicated trading relationships. For Han Chinese, the percentage of friend relationships was only 12.8% for physicians in big hospitals, but 53.5% indicated trading relationships.

### Factors Influencing D-P Relationships

When perceiving relationships with primary care doctors, both for unadjusted OR and adjusted OR, Han Chinese are more likely to reflect trading-type or other types of relationships than friendships. However, ethnic minorities are more likely to think of the D-P relationship as a friendship than a trading relationship (Nagelkerke's *R*^2^ = 0.146) (Table 5).

**Table 5:** Multinomial regression models for relationship with grass-roots doctors.

**Independent variable**	**Unadjusted OR withgayu [95% CI]**	**Adjusted OR withgayu [95% CI]**
Age group (Reference: 65–)		
Other types 16+		
	0.600 [0.402, 0.895]	0.645 [0.408, 1.020]
Education (Reference: ≥Undergraduate)		
Trading/reciprocal-type		
Never attend any school	0.354 [0.254, 0.495]	0.603 [0.403, 0.904]
Primary school	0.742 [0.554, 0.994]	0.978 [0.692, 1.382]
Other types		
Never attend any school	0.413 [0.290, 0.586]	1.053 [0.684, 1.623]
Primary school	0.721 [0.527, 0.984]	1.095 [0.754, 1.592]
Income (Reference: quartile 4)		
Trading/reciprocal-type		
Quartile 1	0.339 [0.275, 0.419]	0.408 [0.320, 0.520]
Quartile 2	0.739 [0.601, 0.909]	0.826 [0.659, 1.036]
Quartile 3	1.633 [1.309, 2.039]	1.673 [1.323, 2.116]
Other types		
Quartile 1	0.361 [0.293, 0.466]	0.388 [0.304, 0.496]
Quartile 2	0.519 [0.418, 0.644]	0.567 [0.447, 0.721]
Quartile 3	0.727 [0.571, 0.927]	0.742 [0.573, 0.961]
Province (Reference: Qinghai)		
Trading/reciprocal-type	1.327 [1.148, 1.536]	1.019 [0.858, 1.209]
Other types	3.057 [2.584, 3.617]	2.405 [1.979, 2.923]
Ethnicity (Reference: Minority)		
Trading/reciprocal-type	3.683 [2.912, 4.658]	2.547 [1.951, 3.326]
Other types	5.230 [3.907, 7.001]	2.345 [1.693, 3.249]

*OR, odds ratio; CI, confidence interval. Reference group: friend-type*.

When perceiving relationships with doctors in large hospitals, this ethnic difference is also evident. Ethnic minorities' tendency to have friendly D-P relationships is consistent with lower levels of education, lower income, and provinces that are economically underdeveloped (Qinghai Province vs. Zhejiang Province) (Nagelkerke's *R*^2^ = 0.160) (Table 6).

**Table 6:** Multinomial regression models for relationship with doctors in large hospital.

**Independent variable**	**Unadjusted OR withgayu [95% CI]**	**Adjusted OR withgayu [95% CI]**
Education (Reference: ≥Undergraduate)		
Trading/reciprocal-type never attend any school		
	0.299 [0.203, 0.440]	0.568 [0.349, 0.922]
Other types		
Never attend any school	0.324 [0.217, 0.483]	0.811 [0.489, 1.344]
Primary school	0.585 [0.400, 0.856]	0.823 [0.521, 1.301]
Income (Reference: quartile 4)		
Trading/reciprocal -type vs. friends		
Quartile 1	0.272 [0.213, 0.347]	0.387 [0.290, 0.516]
Quartile 2	0.578 [0.448, 0.744]	0.668 [0.503, 0.887]
Quartile 3	1.464 [1.090, 1.967]	1.461 [1.065, 2.004]
Other types vs. friends		
Quartile 1 quartile 2	0.349 [0.271, 0.448]gayu 0.636 [0.474, 0.854]	0.487 [0.362, 0.656]gayu 0.582 [0.432, 0.785]
Province (Reference: Qinghai)		
Trading/reciprocal -type	1.316 [1.111, 1.559]	0.598 [0.476, 0.751]
Other types	2.327 [1.929, 2.807]	1.109 [0.869, 1.413]
Ethnicity (Reference: Minority)		
Trading/reciprocal -type	7.335 [5.819, 9.247]	6.931 [5.206, 9.226]
Other types	8.164 [6.195, 10.759]	6.503 [4.683, 9.030]

*OR, odds ratio; CI, confidence interval. Reference group: friend-type*.

### Interactions

**Figure 1:**
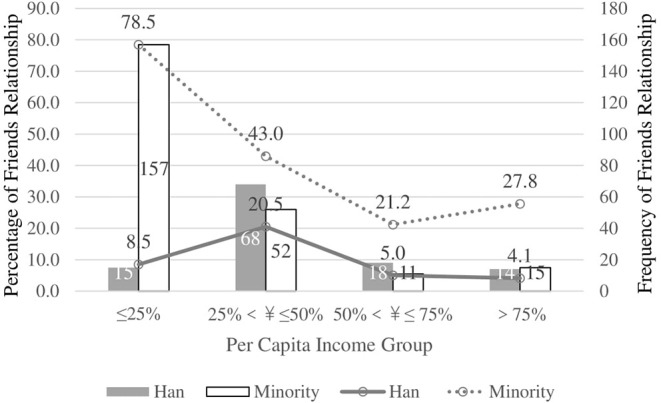
Interaction between ethnics and income for Friends relationship with doctors in Primary Care Institution in Qinghai Province.

When ethnicity was added to the model, the significant unadjusted OR of trading-type vs. friendship with physicians in different provinces become insignificant (primary care) or reversed (big hospital) in an adjusted OR (Tables 5, 6). To better understand the influence of ethnicity, income, and/or province on the perception of D-P relationships, we ran interaction tests for these factors. First, we tested ethnicity and income for primary care in Qinghai Province, and added gender, age, and education as predictors for different types of D-P relationships (χ^2^ = 469.70, *p* < 0.001, Nagelkerke's *R*^2^ = 0.124) (Figure 1).

**Figure 2:**
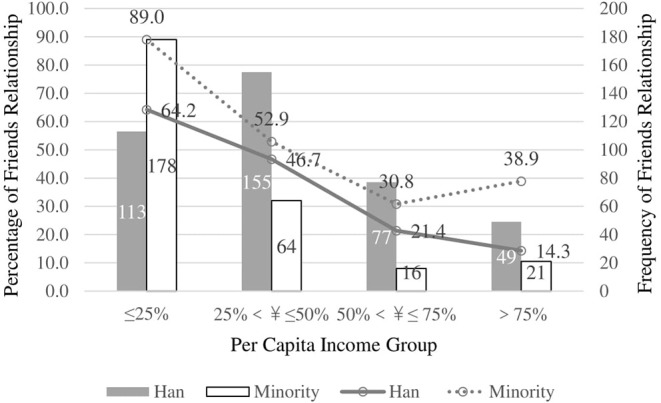
Interaction between ethnics and income for Friends relationship with doctors in Big Hospitals in Qinghai Province.

The interaction between ethnicity and income for relationships with doctors in big hospitals in Qinghai Province is also significant (χ^2^ = 606.00, *p* < 0.001, Nagelkerke's *R*^2^ = 0.359). Ethnic minorities in Qinghai Province are more likely to perceive doctors in big hospitals as friends when they belong to a lower economic group (Figure 2).

**Figure 3:**
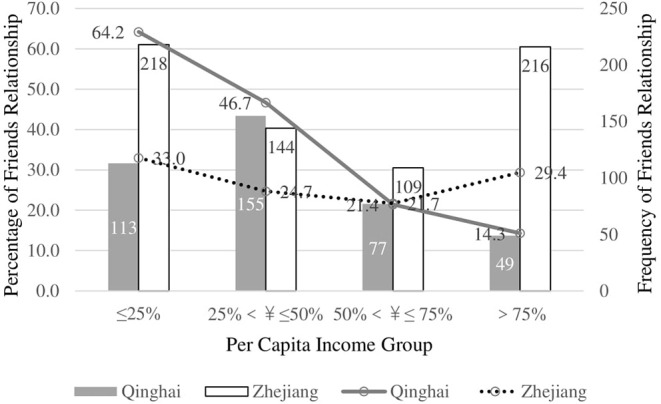
Interaction between ethnics and income for Friends relationship with doctors in Primary Care Institution for Han Chinese.

Since minorities in Zhejiang Province are few (27 people), we tested the interaction of province and income for friend D-P relationships in primary care institutions for Han Chinese only, showing significance (χ^2^ = 374.10, *p* < 0.001, Nagelkerke's *R*^2^ = 0.109). Income has no influence on the type of D-P relationships with primary care doctors in Zhejiang Province. However, as economic status rises, people in Qinghai Province are less likely to have friendly D-P relationship with primary care doctors (Figure 3).

**Figure 4:**
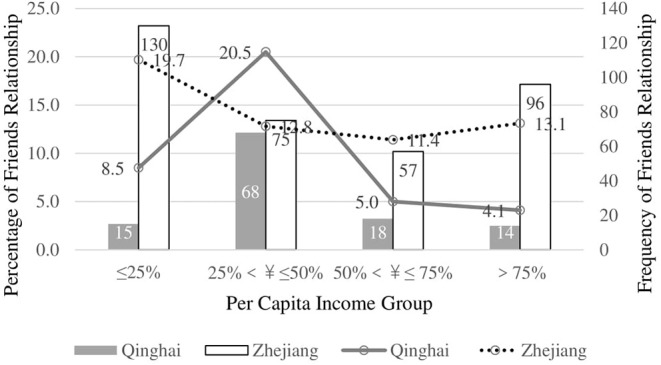
Interaction between ethnics and income for Friends relationship with doctors in Big Hospitals for Han Chinese.

The impacts of ethnicity on D-P relationships in big hospitals is also significant (χ^2^ = 256.15, *p* < 0.001, Nagelkerke's *R*^2^ = 0.079) (Figure 4). We compared province and ethnicity with people's first choice of health institution with two chi-square tests. The results show that people in Qinghai Province are more likely to go to a primary care institution (82.7%), while those in Zhejiang Province tend to go to big hospitals (44.5%) (χ^2^ = 335.90, *p* < 0.001, μ^2^ = 0.28). There is no difference between Han Chinese and ethnic minorities in regard to first contacted health institution *(*χ^2^ = 1.03, *p* > 0.05, μ^2^ = 0.01).

## Discussion

The current study advances our understanding that D-P relationships vary according to ethnicity and socioeconomic status. Ethnic minorities are most likely to perceive their physicians as *friends*, while Han Chinese are more likely to perceive a trading relationship with their physicians. The frequency of friendly D-P relationships is consistent with lower levels of education and income. This pattern is evident regardless of province or type of health care institution and seems to be correlated only with socioeconomic status. Ethnic minority groups have lower socioeconomic status than the Han Chinese, and Qinghai Province has a significantly lower GDP than Zhejiang Province.

### Courtesy Calls for Help From the Wild

While socioeconomic status impacts D-P relationships, there is also a correlation with history and cultural transitions among these groups. The culture in which individuals live influences the way they communicate (48). As an important form of communication, the D-P relationships vary according to culture related to ethnicity. Our results are consistent with the previous Israeli D-P relationship study (5), that compared ethics, age, gender, education, income, doctor visits, and health status, finds there is a most robust difference in the D-P relationships between Arabs and Jews. Relatively more Arabs (53.8%) than Jews (21.6%) reported friend relationships. In Israel, Arabs compose the minority, and Jews represent the majority ethnic group (6,377,000 out of 8,550,000). The Uyghur, the second largest minority group (33.1%) in our study, predominantly Muslim, reflect similar D-P relationships to the Arabs in the Israeli study.

The impact of ethnicity on D-P relationships in China can be interpreted in light of the Confucian saying, *Courtesy calls for help from the wild* (礼失而求诸野) (45). The dominant school of Chinese ethics is Confucianism. In contrast to the self-focused traditions of the West, Confucian influence on East Asian cultures leads individuals to consider others explicitly, stressing the notion that productive societies should have a relationship- and group-centered focus (49).

Confucianism considers four hierarchical relationships as the basis of society: king and subject, father and son, husband and wife, and elder and younger brother. In each of these relationships, the latter party with less power is expected to conform to the ruling of the more powerful party (50). Confucianism has been more strongly retained in rural areas of China than in urban areas. As Fei (51) noted, rural China is an acquaintance society that is regulated by village rules (51). Following this pattern, D-P relationships are more likely to approach friend relationships in rural areas than in urban hospitals.

China has experienced significantly economic reforms. Capitalist markets have been accompanied by western culture and urbanism; as a result, traditional Confucianism has partly collapsed, especially in areas strongly influenced by economic reform, such as the eastern areas where the Han Chinese are concentrated. However, over the course of its history, predominant Chinese culture has exchanged and communicated with the surrounding ethnic minorities, who play roles of competitors, supervisors, and enhancers of Chinese culture. There is a dynamic system of interaction between Chinese central culture and the cultures of surrounded ethnic minorities. In many cases, the minority ethnic groups inherited and recreated elements of traditional Chinese culture when the central regions suffered wars, decline, and mourning. Subsequently during revitalization, the political power of the Han Chinese would learn and re-inherit these traditions from the ethnic minorities. This process is known as “seeking from the surrounding ethnic minorities when the rite was lost (of the central culture),” or *courtesy calls for help from the wild*.

Three decades ago, contrary to current strained D-P relationships, Chinese patients trusted their doctors so much that they were unfamiliar with informed consent. What the doctor did was regarded as representing the best interests of the patient, and the doctor thought it only right to make decisions for the patient (52). However, today violence in Chinese hospitals is not uncommon. In 2015, a poll conducted by the Chinese Medical Doctor Association found that 13 % of 12,600 doctors said they had been physically assaulted by their patients in the last year (53). Despite the number of lawsuits in 2016 falling by 6.7 %, the negative effects of individual cases was still a significant issue (54).

Because of limited modernization, the value of traditional culture in delayed market-oriented reform is significant. The pursuit of utility is increasingly displacing the efforts to retain tradition, is exacerbated by the absence of religion, psychological confusion, and the mutation of social ethics. However, due to the limited influence of modernization, Chinese western minority areas maintain relatively traditional and straightforward values and religious beliefs. Thus, they are more likely to treat the D-P relationships as friends instead of trading-relationship.

### Income Gap

As Louis Wirth suggested, urbanism brought social disorganization and individual alienation (55). It congregated “critical masses” or people to threaten the social trust. After the Second World War and the Civil War, the feudal social and political system in the western national regions not only ended, but the economic and cultural system of the former society also disintegrated. In effort to modernize, the government established a highly integrated network of organizations to undertake economic, social, and cultural organization.

Although culture and ethnicity play a major role in D-P relationships, perceived D-P relationships of primary care institutions also demonstrate the influence of economy. Social psychologists have demonstrated that people who strongly value money have poorer relationships (56), and money can injure social relationships (57). In our study, the friendly D-P relationships of Han Chinese in Qinghai Province are not very sensitive to income. As shown in Figure 1, however, friendly D-P relationships among ethnic minorities in Qinghai Province decrease as income increases. Moreover, findings among Han Chinese demonstrate the power of income gaps (Figure 3). The standard deviation of income in Qinghai Province (24,454 ¥) is higher than in Zhejiang Province (21,625 ¥). This shows that income is not correlated with D-P relationships in Zhejiang Province. However, in Qinghai Province, lower income groups are more likely to perceive D-P relationship as friends than higher income groups. The highest group (quartile 4) is the least likely to perceive a friendly D-P relationship.

### Levels of Health Institution

Given the diversity of the country, the overarching goal for Chinese public hospitals, is to recognize that different models are needed for different geographic locations and hospital settings (41). This is in contrast to hospital reform in USA and Europe, which focus on cost control. Specific management plans for service stations, health hospitals, and big hospitals should be different.

Chinese big hospitals are concentrated in cities but the primary care institutions are scattered around the rural areas, which is just consistent with the proportion of Han physicians to ethnic minority physicians, especially in ethnic autonomous prefectures in Qinghai. In our interview with a local health official, we have learnt that the ethnic minority village doctors are <10% in areas where Han Chinese major live. However, the ethnic minority village doctors account for 90% of the village doctors in ethnic autonomous prefectures. In township health center, there are still about 60% physicians are ethnic minorities. As a result, patients have a higher opportunity to contact with ethnic minority physicians in Qinghai ethnic minority rural areas than in big cities. The ethnic minority physicians in rural areas, as we have discussed above, who have preserved Chinese traditional personality of warm and friendly, are more salient than in urban Han Chinese physicians. Thus, they were more likely to be perceived as friends by their patients than big hospital physicians.

The proportion of ethnic minority residents in rural areas is also more significant than the index of urban areas. Researchers have demonstrated that the people who live in more densely populated areas tend to report less satisfied interpersonal communication because high population density implied worse resources scarcity and more furious competition (58–60). However, the population density in rural areas is less than in urban cities. Thus, ethnic minority residents have more opportunity to know about each other, especially their ethnic minority physicians.

According to the social psychological theory, similarity, familiarity, proximity, and to be liked is the basic rules in interpersonal attraction (61). Similarity indicated that people tend to make friends who are similar to themselves in demographics and characteristics, in which ethnicity is a very important factor. Perceived similarity results in the assumption that people have more in common, facilitating warmer, more comfortable interactions, all of which facilitate attraction. People are more likely to make friends with people who has the same race or ethnicity with them (62). Thus, the distribution of ethnicity minorities and Han Chinese of physicians in rural and urban areas could explain patients' preference of perceived D-P relationships among different levels of health institutions: patients like to make friends with ethnic minority physicians who share the same characteristics with themselves in rural areas, but it is difficult for patients to make friends with Han Chinese physicians with significant western or modern characteristics.

As masters of medical technology, doctors are the medical experts. Thus, there is a power relationship between doctors and patients according to social power theory (63, 64), including expert power, referent power, and even legitimate power (65, 66). Powerful people usually stereotype others to save cognitive resources (67), control others (68), and objectify other people (69). Thus, doctors with higher technical abilities (usually found in bigger hospitals) take a leading trading-type role when they communicate with patients. People believe the best way to enhance effectiveness is to fit the right kind of leaders to the situations they face (70). As a service occupation, medical practice provides services of different grades to meet patient expectations and results in the best service effect.

Realizing these facts, the Chinese State Council (71) issued a statement to clarify the function of medical institutions at all levels, indicating that medical and health service at hospitals and primary care institutions should be liable for the treatment of different diseases. Urban tertiary hospitals provide services for critical and complex diseases. Secondary hospitals mainly provide treatment for common diseases, as well as the immediate treatment of the patients with acute, severe, or complicated diseases. Primary care institutions, rehabilitation hospitals, and nursing homes provide treatment, rehabilitation, nursing services for stable patients with chronic diseases, the elderly, and patients with advanced cancer (72).The cross-sectional design of our study makes inferences of causal relationships between indicators of D-P relationships and associated factors impossible. Our study site of developed province (Zhejiang) has a deeper degree of globalization, modernization, and urbanization than undeveloped province (Qinghai) because of the geographic and socioeconomic conditions. Globalization, modernization, and urbanization has broken up the Chinese traditional culture. As a result, they provided conditions for ethnic minorities to preserved more friendly relationship with physicians than Han Chinese. The effect which globalization has played may be as same as higher socioeconomic status, higher income, and higher hierarchy of hospitals. WHO has proposed “health policies should be culturally appropriate” as an approach of many other public health studies (73–76). Globalization could explain why big hospitals, most of which are mainly tertiary hospitals in the cities, are more likely to perceive trading type relationship, but it could not explain why ethnic minorities more likely to perceive friendly relationship. Only “courtesy calls for help from the wild” and globalization are considered at the same time, can they be explained together about the opposite perception of D-P relationship between ethnic minorities and Han Chinese. Further studies should adopt qualitative research in anthropology, to explore the impacts of Chinese Confucian culture and the tide of globalization.

## Conclusion

The primary contribution of this research is the finding that D-P relationships differ for Han Chinese and other ethnic minorities. Ethnic minorities are most likely to perceive their physicians as friends, while Han Chinese are likely to have a trading relationship with their physicians. Compared to Han Chinese, ethnic minorities retain Confucian ideals, then which results in a focus on friendly relationships and respect of others. This discrepancy is more obvious according to the hierarchy of health institutions. The Han Chinese have experienced a higher degree of globalization, economic reform, and adoption of western values, which has influenced their culture and lead to a tendency to view the D-P relationships as transactional. The bigger hospitals in urban areas also increase this trend. The awareness of these changes and trends in D-P relationships increases an understanding of the increase in dissatisfaction and violence that are presented in today's medical field.

## Data Availability Statement

The raw data supporting the conclusions of this manuscript will be made available by the authors, without undue reservation, to any qualified researcher.

## Ethics Statement

The studies involving human participants were reviewed and approved by the Ethics Committee of Zhejiang University. Written informed consent to participate in this study was provided by the participants' legal guardian/next of kin.

## Author Contributions

QY and HD: study concept and design, and Obtained funding. QY: drafting of the manuscript. QY and MY: statistical analysis. HD: study supervision. All authors critical revision of the manuscript for important intellectual content, acquisition, analysis, and interpretation of data.

### Conflict of Interest

The authors declare that the research was conducted in the absence of any commercial or financial relationships that could be construed as a potential conflict of interest.
